# Measurement of polystyrene photodegradation rate using a quartz crystal microbalance

**DOI:** 10.1049/nbt2.12076

**Published:** 2022-01-08

**Authors:** Abdullah N. Alodhayb

**Affiliations:** ^1^ Department of Physics and Astronomy College of Science, King Saud University Riyadh Saudi Arabia

**Keywords:** chemosensors, crystal‐based sensors, photodegradation, polystyrene, quartz crystal microbalance

## Abstract

Polystyrene is a very popular polymer utilised in the manufacture of various consumer products. This polymer is very cheap; however, after its usage, the slowness of its photodegradation leads to environmental pollution. In this report, the author presents a technique to systematically measure the rate of photodegradation of a thin polystyrene film. The said film was made to coat a quartz crystal microbalance (QCM) sensor. In order to detect polymer degradation and the reduction in the molecular weight, the resonance frequency of the sensor was monitored for 24 h. Results revealed that QCM sensor irradiation with ultraviolet light with a wavelength of 365 nm and optical power of 1.5 mW caused a quite significant change in the polymer structure.

## 1 INTRODUCTION

Polystyrene (PS) is one of the most common plastic materials used in consumer goods, for instance in packaging and food serving. This plastic is also extensively used in industries such as construction, automotive, electronics, medical, and aerospace. Unfortunately, in most contexts, no clear and effective mechanisms exist for PS recycling, so most of this plastic ends up in the oceans and landfills, or it remains in the open atmosphere. Given the widespread use of this material and the lack of an efficient recycling process for it, PS is a substantial contributor to plastic pollution [1]. Evidence also suggests that PS contributes to nanoplastic pollution, which results in nanoplastics contaminating drinking water and the food supply chain [2].

Notably, multiple techniques have been reported for the recycling of PS waste [3]. Hearon as well as Noguchi reported D‐limonene as a solvent to be used in the modification of the composition of the PS molecular chain [4, 5, 6]. Shah reported the photodegradation of PS achieved by exposure to ultraviolet (UV) light, in a study whereby the researchers continuously monitored over several minutes, the viscosity change of the material exposed to UV light irradiation [7]. Another study reported that the UV light causes photooxidative degradation that causes breakage of polymer chains, which reduces the molecular weight of the thin film [8]. Arandes reported a process of thermal recycling of PS that relied on light cycle oil [9]. In spite of their respective advantages and disadvantages, all such techniques displayed very good promise, so far, their efficacies have not been studied at a scale of a micro‐level thin film. Thakur et al. discussed the various methods of recycling PS‐based plastics including landfilling, and chemical and mechanical recycling [10]. Gutiérrez et al. reported the dissolution technique as the primary step in PS recycling [11]. The main advantage of this technique is its cost‐effectiveness, with the main disadvantage being the impossibility to achieve polymer precipitation at room temperature. Achilias et al. proposed PS chemical recycling by way of pyrolysis [12]. This technique involves the use of the liquid by‐product released as a result of pyrolysis as raw material for the reproduction of PS. The main advantage of the process is that it affords the reuse of the styrene monomer released by pyrolysis. Its disadvantage consists of the possibility that aromatic compounds contained in the liquid fraction obtained as part of the process end up acting as chain transfer agents, altering the shape of the reaction rate curve. Yang et al. investigated a solid‐phase photocatalytic degradation of PS conducted over copper phthalocyanine [13]. This technique was performed under the illumination of radioactive fluorescent light in the air. The researchers observed that PS photodegradation resulted in an increase in the rate of PS weight loss, a reduction of the PS molecular weight and of the amount of volatile organic compounds, as well as an increase in the amount of carbon (IV) oxide. The advantage of the described technique consists of the reduced weights and volatility of PS while its disadvantage has to do with the need to carry out sample irradiation in the air.

The main objective of PS recycling is to reduce its carbon footprint. Polystyrene originates from petroleum, which contributes vastly to the carbon footprint across the globe. Recycling PS would extend the lifecycle of the material, and it would reduce the pressure on the available petroleum resources, which in turn would cut down the carbon footprint. Finally, recycling PS is important because it reduces waste, by cutting down the amount of PS going to landfills or ending up in water bodies. The slowness of the process of PS photodegradation contributes to the amount of plastic waste polluting the environment, some of which finds its way to water bodies, where it floats on the water surface [14]. In this article, we report a systematic measurement of a thin film of PS exposed to the UV light for a prolonged period. As it is well known that UV reduces the molecular weight of the plastic, it was very important for us to employ a technique that would be cheap, sensitive, and fast in delivering results [7, 8]. Therefore, we have utilised a quartz crystal microbalance (QCM) that is a well‐known sensor to perform various measurements. Such sensors have been successfully used to monitor biofilm growth, cell adhesion, blood coagulation, and viscosity measurement [15, 16, 17, 18]. QCM has also shown a great potential to be used in environmental, chemical, and medical applications [19, 20, 21, 22]. In this study, with an aim of low cost and rapid response, we exploited high sensitivity and well‐known understanding of a QCM to measure the effect of UV on a thin film of PS on a QCM.

## 2 EXPERIMENTAL

**FIGURE 1:**
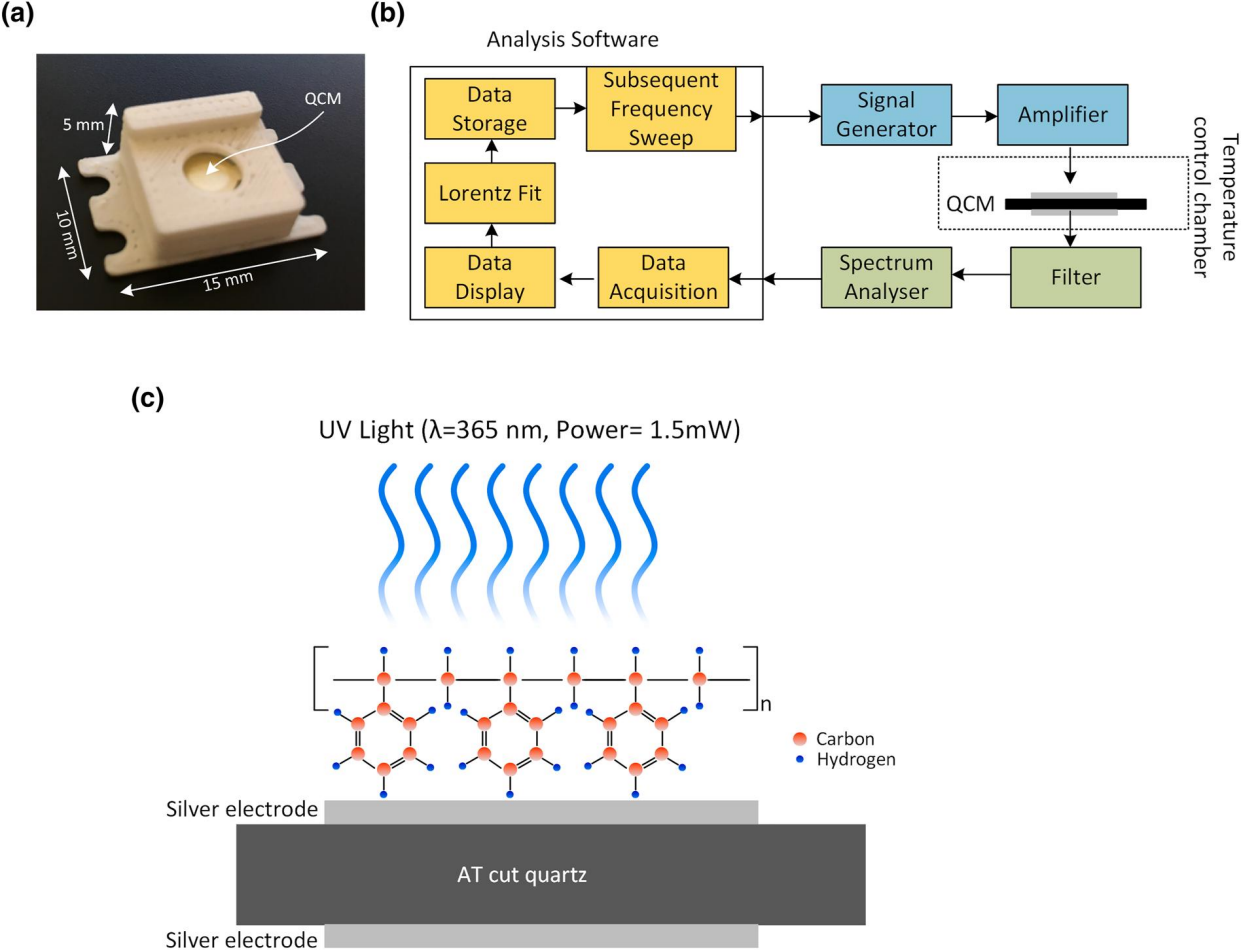
(a) Quartz crystal microbalance (QCM) sensor coated with a polystyrene (PS) thin film. (b) Schematic representation of the measurement instrument. (c) Schematic representation of the QCM sensor ‘decorated’ with a long molecular chain of PS being irradiated with ultraviolet (UV) light

Polystyrene pallets (CH_2_CH[C_6_H_5_])_*n*_ with a molecular weight of ∼280,000 were acquired from Sigma Aldrich. The pallets were characterised by having an amorphous structure, so it was important to prepare a liquid solution that could be deposited on a QCM on a thin film. Anhydrous toluene (99.8%, Sigma Aldrich) was used as a solvent to dissolve the PS pallets. Specifically, 2 g of pallets were added to 1 ml of toluene. In order to ensure complete pallet dissolution and obtain a homogeneous solution, the mixture thus obtained was heated at 45°C for 2 h. For these experiments, the QCM sensors (QD8) were acquired from Fourien (Edmonton, Canada) (Figure 1a). The sensors (diameter: 8 mm) were pre‐packaged for convenient handling; we carefully avoided any dust contamination of the sensors and ensured their compatibility with the QCM. The sensor package was made up of a plastic called acrylonitrile butadiene styrene.

To deposit a thin film on a QCM sensor, the sensor package was mounted on a spin coater using a double‐sided tape. Subsequently, a 50‐μL droplet of a toluene/PS mixture was placed in the centre of the QCM sensor using a pipette. The sensor was then spun at 2500 rpm for 60 s. This process resulted in the production of a thin film with a thickness of less than 1 μm.

The QCM sensors mechanically resonate in response to an electrical excitation signal that matches their natural resonance frequency. With every resonance cycle, the quartz material generates a sinusoidal current. In order to exploit the QCMs for sensing applications, we used low‐noise and high‐speed electronics to record the resonance frequency of the sensors. Figure 1b depicts the working schematic of the measurement instrument. First of all, using the data acquisition and analysis software, the excitation frequency was swept with a specific range where the effort was made to keep the resonance frequency in the centre. Therefore, in order to perform the measurements with high accuracy and repeatability, a commercial QCM measurement instrument was acquired from Fourien Inc. (Edmonton Canada). The instrument has a built‐in signal generator that after amplification electrically excites the QCM sensors. The sensor comprises two silver‐coated electrodes on either side of it. The electrical response of the sensor is monitored using the feedback module of the instrument. The data is passed from the band‐pass filter so that any high‐frequency or low‐frequency noise should be rejected. Afterwards, the built‐in spectrum analyser performs a fast Fourier transform on each signal.

Note that in Figure 1c, a cross‐sectional view of quartz coated with a PS film is depicted. In order to study the photodegradation of the PS film by UV light irradiation, the QCM sensor was exposed to irradiation with the UV light at a wavelength of 365 nm and an optical power of 1.5 mW.

## 3 RESULTS AND DISCUSSION

Before any deposition on the sensor, its resonance frequency was determined by sweeping an excitation sinusoidal frequency from 3.99618 to 4.0024 MHz. By this approach, the resonance frequency of the sensor was identified to have a value at 3.999590 MHz. The spectrum for the resonance frequency as well as the phase was determined using the frequency sweep resolution to be 2 Hz, the averaging factor 128, and the frequency dwell cycles 500. These data enabled us to complete each sweep within 70 s. In order to evaluate sensor stability, the measurement of the uncoated QCM was carried out continuously for 14 h. To determine the standard deviation of the measurements, data for 14 min was used to calculate the value for the standard deviation (17 Hz).

**FIGURE 2:**
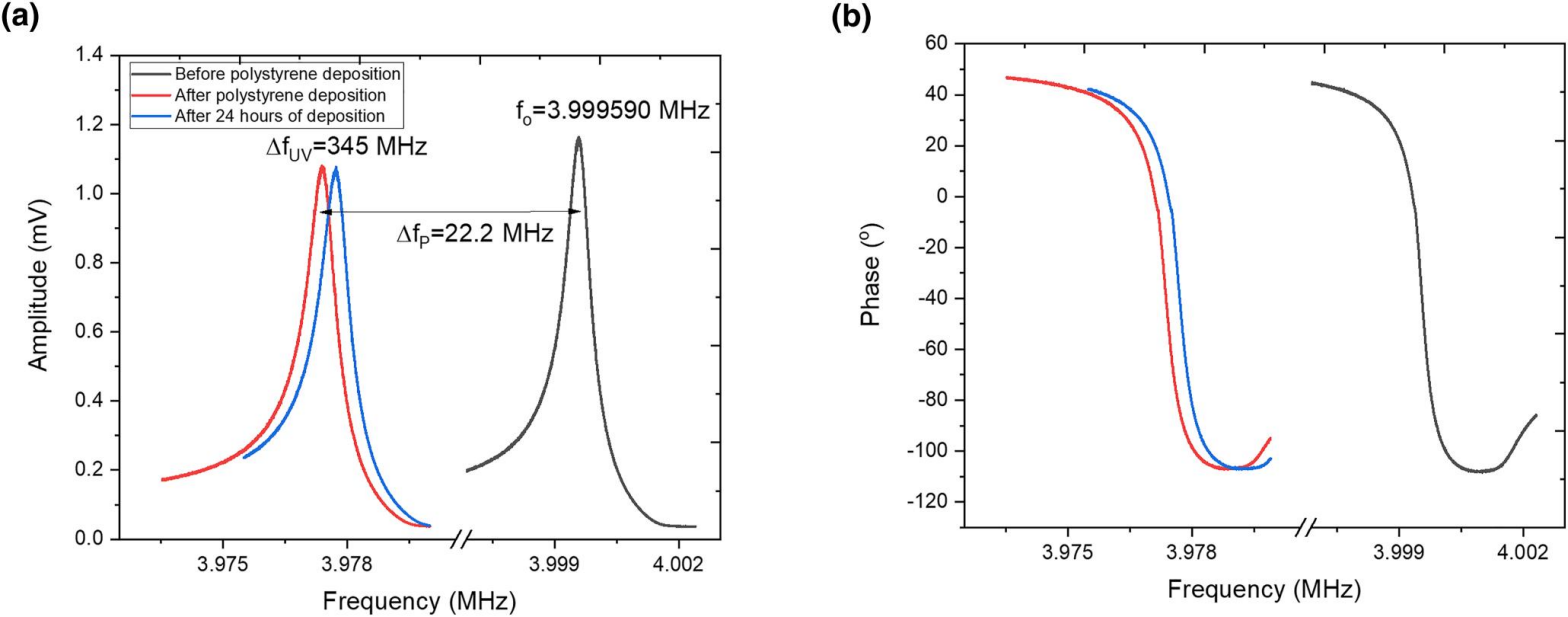
(a) Resonance frequency and (b) phase and spectrum of quartz crystal microbalance (QCM) sensors, either uncoated (black curves) or coated with polystyrene (PS) (red and blue curves)

After conducting measurements on the blank (uncoated) QCM, the sensor was coated with PS (as described in the Experimental Section). After allowing the solvent to evaporate to dryness, the sensor's resonance frequency was determined. The frequency sweep of a sinusoidal signal was performed from 3.97353 to 3.97999 MHz. The other measurement parameters were kept the same as in the measurements conducted on the uncoated sensor. Results from the measurement procedure indicated that due to the increase in mass associated with the addition of the PS thin film, the resonance frequency of the sensor had dropped by 22.2 kHz (see Figure 2a). This change in resonance frequency also demonstrated that the thin film had achieved good coupling with the surface of the sensor.

**FIGURE 3:**
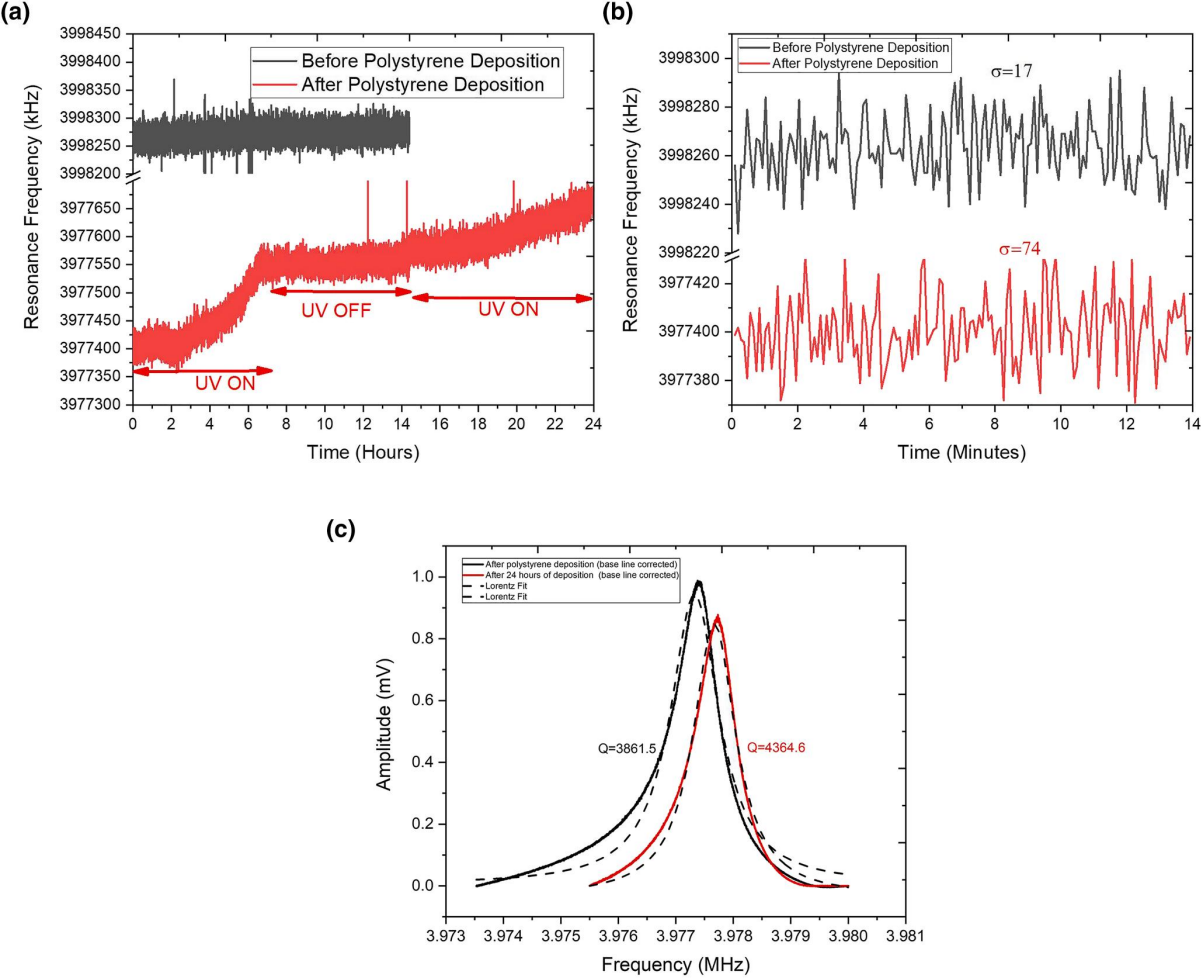
(a) Measurement of the resonance frequency of quartz crystal microbalance (QCM) performed for an extended time period under periodic ultraviolet light irradiation. (b) Standard deviation (*σ*) calculation on a subset of the data. (c) The calculation of quality factor (*Q*)

Subsequently, in order to perform the photodegradation of the polymer film, the sensor was irradiated with the UV light at a wavelength of 365 nm and at an optical intensity of 1.5 mW, using the integrated UV source in the instrument. Data were recorded continuously for 24 h: the UV light was first kept on for 7 h, it was then kept off for 7 h, and, finally, switched on again for 10 h (see Figure 3a). The results of this experiment indicated that over the first 7‐h interval of continuous exposure to the UV light, there was a significant change of about 155 Hz in the resonance frequency of the sensor. This observation can be explained through the photodegradation of the thin layer of the PS polymer. In fact, as a result of its photooxidative degradation, the polymer chain is broken, thus producing free radicals. Photodegradation results in a reduction of the PS molecular weight, which is made evident by the increase in the resonance frequency associated with mass unloading [23].

After the first 7‐h exposure, the UV light was turned off while the measurement of the resonance frequency continued. After carrying on the measurement of the resonance frequency for another 7 h, it was found that the said parameter had not changed in value. This observation descended from the fact that in the absence of UV irradiation, the film had remained intact. After about 7 h of no exposure, the UV light was turned on once again while the measurement was being continuously performed. Once again, a reduction of about 100 Hz in the resonance frequency was measured. This observation was once again evidenced that as soon as the UV light was turned on, the slow process of PS photodegradation resumed.

In order to compare the measurements performed on the uncoated and coated sensor, the standard deviation of the subset of the data reported in Figure 3b was calculated. Evidence indicated that the standard deviation of the measurements conducted on the coated sensor was significantly higher than its counterpart for the measurements conducted on the uncoated one. This difference was possibly due to the degradation activity on the surface of the sensor, which might have caused higher variations in the resonance frequency. The other factors contributing to this instability may also be related to higher thickness as well as any instability in the thin film.

Figure 3c shows the calculation of quality factor (*Q*) through a fitting of Lorentzian function on the baseline‐corrected data, presented in Figure 2a. The *Q* changed by 500 before and after UV irradiation. Considering the high sensitivity of a quartz microbalance, this is not a significant change representing the viscoelastic behaviour as the only factor for the frequency change. In the light of the previous literature [7, 22, 23] and the data of the quality factor, it can be inferred that the change in the resonance frequency as well as the *Q* is primarily because of UV‐based degradation of the polymer while the viscoelastic behaviour also played a role in this.

## 4 CONCLUSIONS

Polystyrene photodegradation was systematically measured relying on a highly sensitive sensor, QCM. A thin layer of the polymer was coated on the surface of the sensor. The said sensor was then exposed to the UV light for several hours. Measurement results were indicative of a clear decrease in the molecular weight of the polymer, which is evidence of photodegradation of the thin layer of the polymer along with changes in the viscoelastic behaviour of the thin film of PS. This approach revealed that such experiments can be conducted to study the degradation of multiple different polymers at the industrial level. Therefore, such polymers can be developed that are characterised by a high rate of UV light‐driven degradation, which would lessen the harm they cause to the environment.

## CONFLICT OF INTEREST

The authors declare no competing interests.

## Data Availability

The data that support the findings of this study are available on request from the corresponding author. The data are not publicly available due to privacy or ethical restrictions.

## References

[1] Kwon, B.G. , et al.: Global styrene oligomers monitoring as new chemical contamination from polystyrene plastic marine pollution. J. Hazard. Mater. 300, 359–367 (2015) 26218303 10.1016/j.jhazmat.2015.07.039

[2] Bojic, S. , et al.: Platform to study intracellular polystyrene nanoplastic pollution and clinical outcomes. Stem Cell. 38(10), 1321–1325 (2020) 10.1002/stem.3244 32614127

[3] Maharana, T. , Negi, Y.S. , Mohanty, B. : Recycling of polystyrene. Polym. Plast. Technol. Eng. 467, 729‐736 (2007)

[4] Hearon, K. , et al.: A high‐performance recycling solution for polystyrene achieved by the synthesis of renewable poly(thioether) networks derived from d‐limonene. Adv. Mater. 2610, 1552–1558 (2014) 10.1002/adma.201304370 PMC4000729 24249666

[5] Noguchi, T. , et al.: A new recycling system for expanded polystyrene using a natural solvent. Part 1. A new recycling technique. Packag. Technol. Sci: Int. J. 111, 19–27 (1998)

[6] Hardjono, H. , et al.: D‐limonene from orange (Citrus Maxima) peel extraction as destructive agent of styrofoam waste. In: IOP Conference Series: Materials Science and Engineering, vol. 1073. no. 1. IOP Publishing (2021)

[7] Shah, S.S. , Ahmad, I. , Ishaq, M. : Degradation study of used polystyrene with UV irradiation. Adv. Mater. Sci. 2, 1–6 (2017)

[8] Yousif, E. , Raghad, H. : Photodegradation and photostabilization of polymers, especially polystyren. SpringerPlus. 2(1), 1–32 (2013) 10.1186/2193-1801-2-398 PMC4320144 25674392

[9] Arandes, J.M. , et al.: Ind. Eng. Chem. Res. 42(16), 3700–3710 (2013)

[10] Thakur, S. , et al.: Recent developments in recycling of polystyrene based plastics. Curr. Opin. Green Sustain. Chem. 13, 32–38 (2018)

[11] Gutierrez Muñoz, C. , et al.: The selective dissolution technique as initial step for polystyrene recycling. Waste Biomass Valoriz. 41, 29‐36 (2013)

[12] Achilias, D.S. , et al.: Chemical recycling of polystyrene by pyrolysis: potential use of the liquid product for the reproduction of polymer. Macromol. Mater. Eng. 292(8), 923‐934 (2007)

[13] Yang, C. , et al.: High photocatalytic degradation activity of the polyvinyl chloride (PVC)–vitamin C (VC)–TiO2 nano‐composite film. J. Hazard Mater. 1783(1–3), 152‐156 (2010) 10.1016/j.jhazmat.2010.01.056 20138426

[14] Stuart, R. , Evans, D. : The environmental effect of reusing and recycling a plastic‐based packaging system. J. Clean. Prod. 11(5), 561‐571 (2003)

[15] David, N. , et al.: Long‐term, on‐line monitoring of microbial biofilms using a quartz crystal microbalance. Anal. Chem. 65(1), 65–69 (1993)

[16] Joachim, W. , Janshoff, A. , Galla, H. : Cell adhesion monitoring using a quartz crystal microbalance: comparative analysis of different mammalian cell lines. Eur. Biophys. J. 28(1), 26–37 (1998) 10.1007/s002490050180 9933921

[17] Marcus, A. , et al.: Quartz crystal microbalance‐with dissipation monitoring (QCM‐D) for real time measurements of blood coagulation density and immune complement activation on artificial surfaces. Biosens. Bioelectron. 21(1), 79–86 (2005) 15967354 10.1016/j.bios.2004.09.026

[18] Ash, D. , et al.: Viscosity measurement of industrial oils using the droplet quartz crystal microbalance. Meas. Sci. Technol. 14(11 **)**, 1955‐1962 (2003)Crossref

[19] Kuchmenko, T. , Lvova, L. : A perspective on recent advances in piezoelectric chemical sensors for environmental monitoring and foodstuffs analysis. Chemosensors. 7(3), 39 (2019)

[20] Ma, X. , et al.: Oriented surface epitope imprinted polymer‐based quartz crystal microbalance sensor for cytochrome c. Talanta. 191, 222–228 (2019) 30262054 10.1016/j.talanta.2018.08.079

[21] Pandey, L. : Design of engineered surfaces for prospective detection of SARS‐CoV‐2 using quartz crystal microbalance‐based techniques. Expet. Rev. Proteonom. 17(6), 425–432 (2020) 10.1080/14789450.2020.1794831 32654533

[22] Rotake, D. , et al.: Highly selective sensor for the detection of Hg 2+ ions using homocysteine functionalised quartz crystal microbalance with cross‐linked pyridinedicarboxylic acid. IET Nanobiotechnol. 14(7), 563–573 (2020) 33010131 10.1049/iet-nbt.2020.0109 PMC8676536

[23] Tryon, J.M. , Achhammer, B.G. : Study of degradation of polystyrene, using ultraviolet spectrophotometry. J. Res. Natl. Bur. Stand. 51(3) (1953)

